# Intensification, regulation and diversification: The changing face of inland aquaculture in China

**DOI:** 10.1007/s13280-021-01503-3

**Published:** 2021-03-05

**Authors:** Richard Newton, Wenbo Zhang, Zhaoxing Xian, Bruce McAdam, David C. Little

**Affiliations:** 1 Institute of Aquaculture, University of Stirling, Stirling, FK9 4LA UK; 2 Shanghai Ocean University, Shanghai, 201306 China

**Keywords:** Aquaculture, China, Consumption, Diversification, Legislation, Wet markets

## Abstract

Trends in aquatic food consumption were matched against farm production surveys within Hubei province and compared to official production data and statistics. Surveys showed that consumer tastes were changing to a much broader aquatic food menu as their spending power increased. Traditional aquaculture species were becoming less profitable due to reduced profit margins as input costs increased and consumption preferences changed. Consequently, many producers were diversifying their production to meet local demand. Some farmers were also de-intensifying by reducing commercial aquafeed inputs and reverting to more traditional methods of dyke-crop culture to optimise trade-offs between input costs and labour, and manage their risk more effectively. In addition, analysis of local data showed that wholesale changes were occurring to aquaculture production as environmental protection legislation took effect which reduced the growing area for carps considerably.

**Supplementary Information:**

The online version contains supplementary material available at 10.1007/s13280-021-01503-3.

## Introduction

**Fig. 1:**
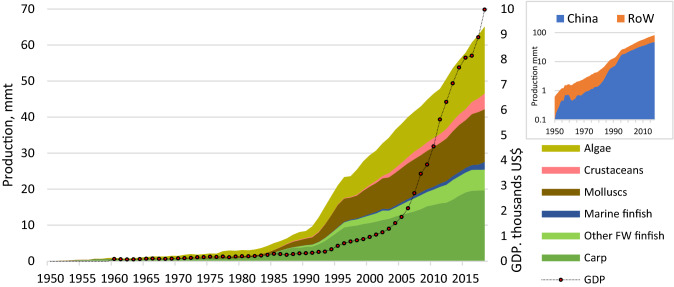
Chinese aquaculture production (million metric tonnes) by major group and GDP per capita. Total Chinese and rest of world (RoW) aquaculture production (log scale). Production data from FAO (2020) and GDP from World Bank (accessed 28/6/2020)

China is the largest aquaculture producer in the world with over 60% of global production by volume. Inland aquaculture has been fundamental to aquaculture growth in China, particularly focussed on traditional carp species that represent over 40% of production (Fig. 1). The contribution of carp to aquaculture is such that seven out of ten of the world’s cultured finfish are carps representing more than half of the 54.3 million tonnes produced in 2018 (Fig. 1) (FAO 2020). According to Tacon and Metian (2015), over 90% of production of fed “Chinese carps”, i.e. grass carp (*Ctenopharyngodon idella*), Wuchang bream (*Megalobrama amblycephala*), black carp (*Mylopharyngodon piceus*), crucian carp (*Carassius carassius*) and common carp (*Cyprinus carpio*) occurs in China. Carp, together with other freshwater finfish, dwarf production of marine finfish and all other aquaculture production categories when edible portion is considered (Edwards et al. 2019).

Increased markets for farmed fish have been linked to both China’s growing population and especially GDP per capita (Fig. 1), in turn fuelling demand for higher value non-staple foods (Crona et al. 2020). Changes in aquaculture production have coincided with large-scale industrialisation, wage growth and urbanisation, leading to growing affluence and purchasing power of Chinese citizens among a powerful emergent middle class (Fabinyi 2011; Chiu et al. 2013). Although it has been long suspected that Chinese fisheries and aquaculture production statistics may have been overestimated (Fabinyi 2011), Chinese government provincial-level data, national and other (derived) statistics at the time of this research suggest that carp production in China had uninterrupted growth (MOA 2016; FAO 2018; Fig. 1).

Despite the volume of Chinese aquaculture production and its increasing global influence, especially in terms of global feed ingredients markets, little has been published internationally about the drivers behind the trends in inland aquaculture from both production and consumption perspectives. In this article, we seek to assess the major changes in the supply and demand for freshwater aquaculture products by a detailed study in Hubei province. Fuelled by demographic, economic and regulatory pressures, we hypothesise that consumer demand for higher value species is leading to a highly diverse and dynamic trajectory of growth emerging in Chinese inland aquaculture. The analysis underpinning this paper falls into three parts. Using Hubei as a case study, we conducted (1) an initial analysis of local secondary production data to show trends in production volumes and land use, (2) internet-based and face-to-face consumer surveys of aquaculture product consumption, and (3) producer surveys of changing production practices.

## Background

Inland Chinese finfish production occurs in a range of different culture systems, particularly in the Central region. Situated between the Yangtze and Pearl River valleys, the region has good access to water through rivers, lakes and reservoir resources. Much of the large-scale expansion and intensification of Chinese aquaculture have occurred in the Central region where both agriculture and industry have faced fewer water resource constraints (Wang et al. 2015). Historically, carp production in China used little or no feed and relied on the natural productivity of the water body (Jia et al. 2013; Wang et al. 2015) which remained the case during the early aquaculture expansion programmes (Fig. 1). However, traditional systems often relied on feeding of grass carp with grass, grown on the dykes of fishponds themselves and on the fertilisation of ponds with organic (and more recently, synthetic) fertilisers to encourage primary production (Ruddle and Zhong 1988; Weiman and Mengqing 2007). Often, fed species such as grass carp or crucian carp were cultured together with lower value filter feeder species such as bighead carp (*Hypophthalmichthys nobilis*) and silver carp (*Hypophthalmichthys molitrix*) where the filter feeders help to maintain suitable water quality.

Until the 1950s fish culture depended on wild capture of fry and juveniles, when artificial breeding of silver carp, bighead carp, black carp and grass carp was achieved (Hishamunda and Subasinghe 2003). Thereafter development was slow, as a result of tightly controlled Chinese government policies that heavily prescribed land use and production though collective ownership, coupled with significant political upheaval during the “Great Leap Forward” and “Cultural Revolution” of the late 1950s into the 1970s (Hishamunda and Subasinghe 2003; Doczi et al. 2014). Subsequent initiatives under Deng Xiaoping to encourage household-level agriculture and pond ownership were originally targeted to promote self-sufficiency and food security following years of famine and extreme hardship amongst the rural poor (Hishamunda and Subasinghe 2003; Doczi et al. 2014; Jiang 2017).

From the 1980s, the government relaxed their management of production and land ownership in line with broader economic freedoms, which allowed producers much higher control over practices and species produced (Hishamunda and Subasinghe 2003). The Chinese government reduced controls over expansion of land use for aquaculture, leading to a large expansion in the land area devoted to aquaculture (Li et al. 2011). Both marine and freshwater areas and the number of cultured species increased, and lake-based aquaculture was targeted as a promising avenue for development (Hishamunda and Subasinghe 2003; Jia et al. 2013). Many shallow low-lying lakes were converted for aquaculture either by separating sections off, using dykes or creating pens with stakes and netting, whereas deeper lakes and reservoirs were used for cage-based production. An estimated 30% of lakes and reservoirs were used for aquaculture by the end of the century (Jia et al. 2013). Initially pond and pen systems relied on complex polycultures with up to nine species filling various niches within a synergistic system (Edwards 2008a; Wang et al. 2015) particularly centred around “the four domesticated fish”; black carp, grass carp, silver carp and bighead carp, and often integrated with other livestock such as pigs, ducks and others. Market opportunities stimulated intensification based around formulated, factory-made diets that became increasingly available to supplement the natural production capacity of water resources used for aquaculture (Jia et al 2013). In tandem with intensification was both a reduction in the variety and complexity of systems (Edwards 2008a, b, 2012); grass carp became by far the single most cultured species, ahead of silver, bighead, crucian and common carps. In contrast, production of black carp lagged until recently (FAO 2020) probably because of its specialist feed requirement (snails). Overall polyculture systems still dominate, centred around the domestic species, but more intensive culture of fewer, more expensive species are also common (Weiman and Mengqing 2007; Edwards 2012). The rapid rise in intensive production spurred a rapid rise in feed production, largely dependent on imported ingredients such as fishmeal and soybean meal (Weiman and Mengqing 2007; Wang et al. 2018). Intensified feeding became the norm even in previously extensively managed lake systems (Jia et al. 2013). Around 55% of Chinese carp production is now currently estimated to be raised on formulated feeds (Tacon and Metian 2015).

**Fig. 2:**
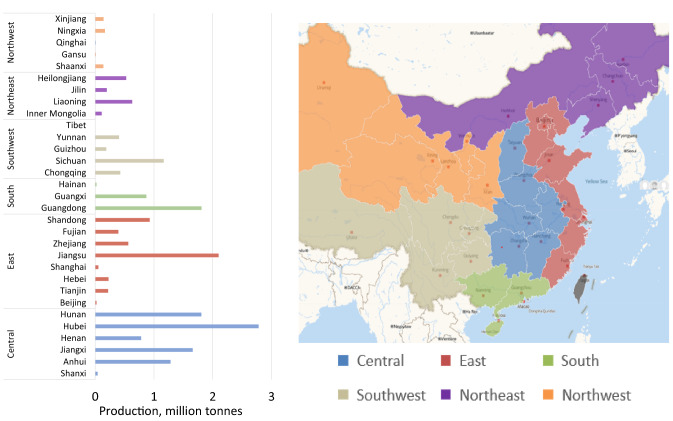
Chinese carp production (tonnes) by province (2018) Data source: MOA (2019). Chinese carp includes grass carp, bighead carp, silver carp, black carp, common carp, crucian carp and Wuchang bream

**Fig. 3:**
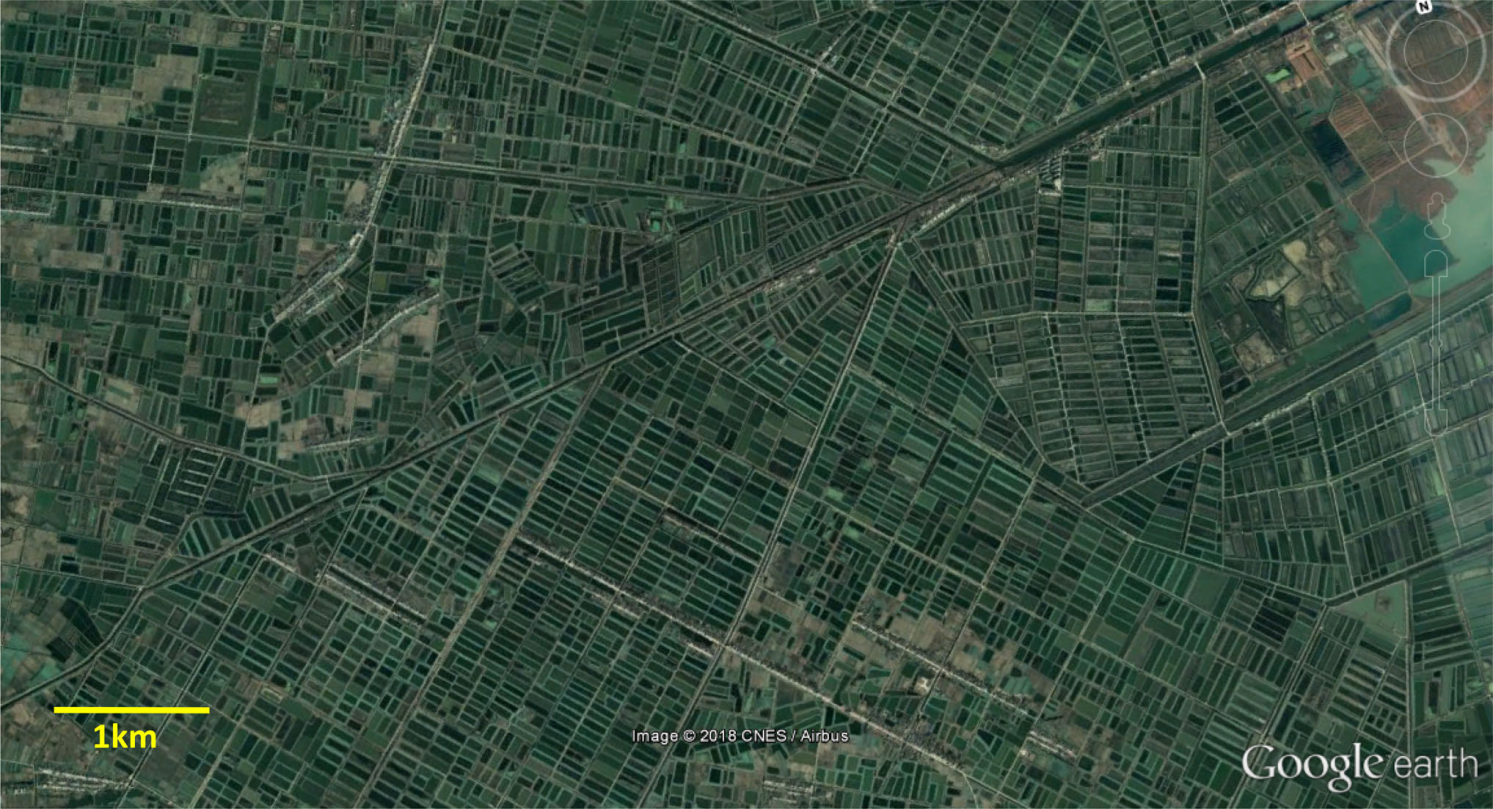
Satellite image of aquaculture ponds in Hubei province (Images courtesy of CNES/ Airbus via Google Earth Pro 2018), 30.27°N 113.16°E

Chinese data indicate that the inland well-watered Central Region is now the heartland of Chinese pond-based carp production with four out of six provinces exceeding a 1 MMT/year production. In particular, Hubei Province ranks as the largest producer of carps in China (Fig. 2), with large swathes of land dedicated to aquaculture (Fig. 3). Hubei provincial data also show large production increases in many other species, suggesting that producers are diversifying away from carp production in favour of higher value species such as red swamp crawfish (*Procambarus clarkii*) to satisfy diverse domestic consumption and for export (Chiu et al. 2013; MOA 2016; Zhang et al. 2017; Wang et al. 2018).

## Materials and methods

### Provincial data analysis

**Fig. 4:**
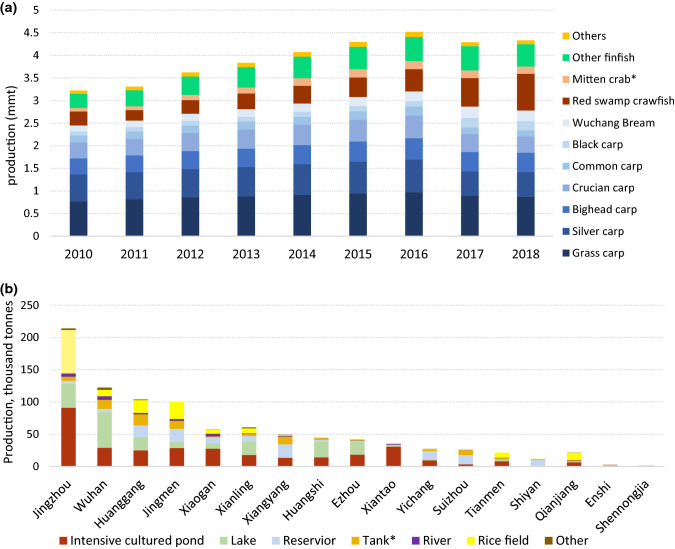
**a** Production of major aquaculture species in Hubei (carp species in blue) 2011–2018 Data source: MOA 2011–2019, **b** Hubei, regional aquaculture production according to system, thousand tonnes. (Hubei Provincial Bureau of Statistics 2016). *Mitten crab (*Eriocheir sinensis*). *A “tank” in this context is a small reservoir used primarily for irrigation, common in highland parts of China

Secondary data from Chinese national and provincial statistics (MOA 2016; Hubei Provincial Bureau of Statistics 2016) were used to identify districts within Hubei province with the largest and most diverse aquaculture production in terms of species and systems. On this basis, the Hubei districts of Wuhan, Jingzhou and Jingmen were selected for survey work, representing the most diverse range of species (Fig. 4a) and culture systems (Fig. 4b) found in Hubei at the time of the survey, as well as offering a mix of urban, peri-urban and rural locations for comparing production and consumption patterns. Survey work was completed in the summer of 2016.

### Consumption surveys

Primary consumer data were collected in two parts based on respondents’ recall. A survey (see Supporting Information) with urban and rural consumers was undertaken to gauge the changing attitudes to seafood consumption in the three districts selected, to complement the production survey (mostly of carp producers), to find trends and changes in carp production practices and the sustainability of the industry. The consumption survey (Supporting Information) assessed past and current habits with regard to consumption and purchase of aquatic products according to different socio-economic groups (referred to as “social status” henceforth). Socio-economic factors were determined using a series of scored indicators (Table 1), based on education level, salary and employment status. Respondents with total scores of 3 or below were considered “low” social status, whereas scores of 4 to 6 were considered “medium” and scores of 7 or more, “high” social status. Rural or urban respondents were determined based on the distance of their home from urban centres (5 km for Jingzhou and Jingmen, and 10 km for the larger city of Wuhan). The consumption survey was implemented by randomly approaching consumers for face-to-face interviews in urban centres around fish markets and other retailers, and electronically using SOJUMP (www.sojump.com), an internet survey tool, after the survey was advertised in local online forums.

**Table 1:** Social demographic indicator scores of consumers in Hubei province

Indicator score	0	1	2	3	4
Education	Below secondary	Secondary	Graduate	Post-graduate	–
Employment	Unemployed, student or retired	Part-time employed	Full time or self-employed	–	–
Annual income	0–40 000 CNY (0–US$ 6200)	40 000–80 000 CNY (US$ 6200–12 400)	80 000–120 000 CNY (US$ 12 400–18 600)	120 000–160 000 CNY (US$ 18 600–24 800)	Above 160 000 CNY (Above US$ 24 800)

### Aquaculture producer survey

A farm production survey (see Supporting Information) was conducted by random sampling of farms in major carp producing areas of Wuhan, Jingzhou and Jingmen identified using Google Earth following a method described by Murray et al. (2014). Questions detailed production methods; species, systems, feed inputs, as well as income and socio-economic factors. Scale was considered one of the most important factors to understanding sustainability of production and the trajectory of change, as small-scale producers are less likely to have access to equitable markets, resources, finance, knowledge and support, with inherently more risk and susceptibility to shock than larger scale producers leading to different and diverse coping strategies (Siar and Sajise 2009; Little et al. 2018; Bush et al. 2019). Scale of production was defined by adopting three scored indicators (Table 2) based on the number of ponds, total culture area and total yield of the farm, where a total score of 2 or less was considered “small scale”, 3 or 4 were considered “medium scale” and a score of 5 or more was considered “large scale”. Feed inputs were often categorised as prepared “farm-mixed” feeds or, more commonly, supplementation with unmixed raw ingredients, such as rice bran, and grass, which have varying moisture contents compared to formulated feeds. Therefore, feed conversion ratios (FCRs) of individual raw ingredients, farm-mixed feeds and formulated feeds were calculated on a dry-weight basis to compare between approaches. In addition to the farm surveys, key informant interviews were conducted at a feed mill, a wholesale market, a privately owned and a government owned hatchery to give contextual information. Statistical analysis was conducted on the farm production data using a standard General Linear Model in Minitab 19 software package.

**Table 2:** Farm-scale indicators for Hubei pond production

Indicator score	0	1	2
Total water area (ha)	< 2	2 to 4	> 4
No. of ponds/cages	1	2 or 3	> 3
Total production per year (tonne)	< 15	15 to 30	> 30

The results of the survey work were compared against current local Ministry of Agriculture data on production volumes, land used for aquaculture in various systems and the total quantity of feed sales. Satellite images of areas under study were then compared over time using Google Earth Pro to gauge land use change around the time of the survey.

## Results

### Secondary data analysis

The trends in carp production can be related to local feed production, as fish production volumes and intensity of production are linked to feeding. However, Chinese national data on feed production are conflicting. China reportedly has over 7000 aquafeed companies, but although national data show China’s total aquatic feed production continued to increase from 16.84 million tonnes in 2011 to 19.30 million tonnes in 2016, a nation-wide Ministry of Agriculture (MOA) feed production monitoring programme based on 180 large-feed companies showed aquatic feed production actually declined from 2.26 million tonnes in 2013 to 1.63 million tonnes in 2016, mainly due to strict water environmental protection regulations and the supply-side reform (National Feed Work Office 2017). Aquatic feed production in Hubei province reportedly increased from 1.72 million tonnes in 2011 (National Feed Work Office 2012) to 2.50 million tonnes in 2016.

**Fig. 5:**
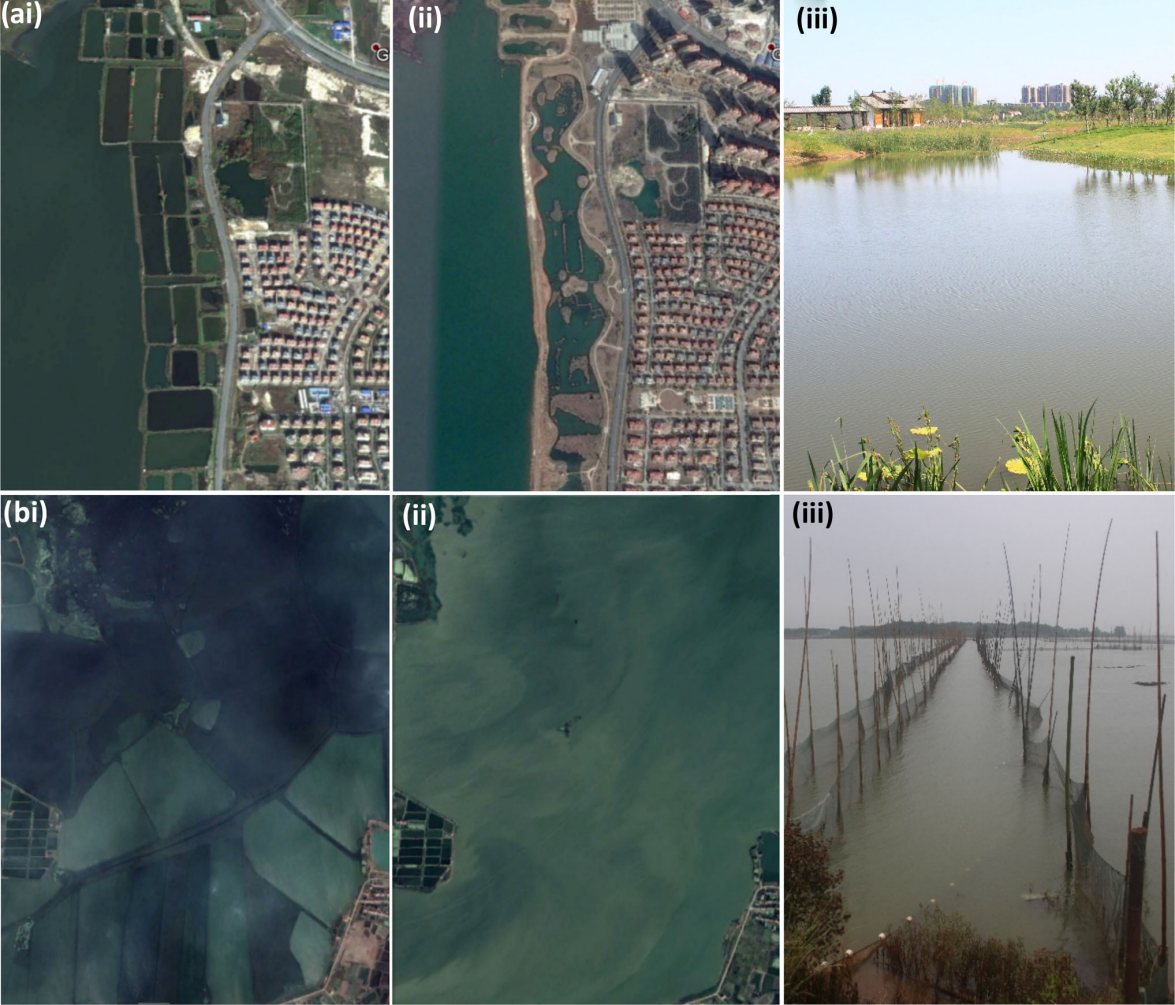
**a** Reclamation of carp ponds due to urbanisation 30.96°N 112.18°E, Jingmen, Hubei. (i) satellite image from 2012, (ii) satellite image from 2016, (iii) photograph taken in 2016. **b** A net pen system, once common, now banned in Hubei, separating areas of a shallow lake for aquaculture, Jingzhou, Hubei, 30.40°N 112.32°E. (i) Satellite image from 2014, (ii) satellite image from 2017, (iii) photograph taken in 2016

Many of the areas that had been identified for surveys were no longer being used for carp production, which only became clear during the farmer survey work (Fig. 5). Environmental regulation has placed constraints on expansion of finfish culture. Since 2011, a series of regulations in China and Hubei have prohibited inputs of feed and fertiliser to lakes and reservoirs, leading to much lower levels of fish production. In addition, further conversion of lakes and other water bodies has also been prohibited, with many being returned to their original state. Therefore, the culture area available for carp culture has been significantly reduced by over 22% from 2014 to 2018, although the reduced area was mainly lake and reservoir areas that had lower yields than ponds (Table 3). Clearly, there is discrepancy between the feed data presented by FAO and MOA with respect to increased carp production due to intensification and expansion compared to the data from the National Feed Work Office and the restrictions imposed on expansion following successive environmental regulations. Far from a continuing upwards trend in carp culture, the evidence suggests that further growth of carp culture is significantly constrained, at least within Hubei Province. This was since confirmed by provincial production data until 2018, presented in Fig. 4a.

**Table 3:** Changes of aquaculture area (ha) in Hubei province (MOA 2015–2019)

	2014	2015	2016	2017	2018
Total	688 000	688 667	698 900	797 575	535 148
Pond	384 405	390 605	414 379	531 167	535 148
Lake	190 068	187 316	179 036	136 662	0
Reservoir	106 641	103 772	98 614	122 584	0
River/Ditch	4600	1002	4403	2813	0
Others	2286	2455	2468	4349	0

### Consumption survey

A total of 267 responses were received from consumer face-to-face interviews and electronic surveys combined (Table 4). More respondents were from the larger urban centres of Wuhan (71.2%) and Jingzhou (20.6%). Jingmen had far fewer respondents in total, mostly classified as rural.

**Table 4:** Number of respondents to seafood consumption survey conducted in Wuhan, Jingzhou and Jingmen

Social status	Urban			Rural			
	Wuhan	Jingzhou	Jingmen	Wuhan	Jingzhou	Jingmen	Total
Lower	66	12	1	38	10	4	131
Medium	61	15	2	10	14	10	112
Higher	11	3	1	4	1	4	24
Total	138	30	4	52	25	18	267

**Fig. 6:**
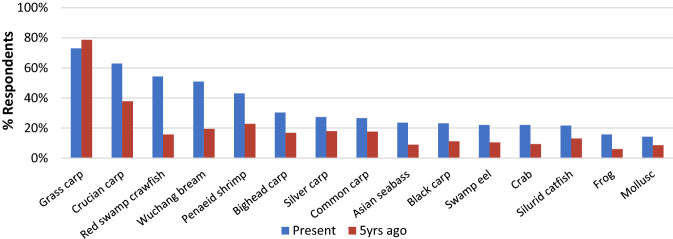
Percentage of respondents consuming seafood species in Hubei province at the time of the survey (over a seven-day recall) relative to most consumed species five years previously (*N* = 267)

Figure 6 shows reported consumption by species and indicates dietary divergence. All carp species were popular among consumers and grass carp remained the most commonly consumed, although the only species that recorded a slight decrease in the last 5 years. The previously dominant position of grass carp in the diet had changed markedly in the last 5 years. While grass carp was consumed by twice as many respondents as any other species five years previous to the survey, crucian carp, crawfish and Wuchang bream were now being consumed by more than half of all consumers on a weekly basis. Consumption of high-value species such as black carp, Wuchang bream (*Megalobrama amblycephala*), Asian seabass (*Lates calcarifer*), Asian swamp eel (*Monopterus albus*) and Chinese mitten crab (*Eriocheir sinensis*) had all more than doubled in frequency compared to 5 years ago, mainly by people of higher social status. However, lower value species such as silver carp also showed an increase in consumption over the same period and were consumed at similar levels across social classes.

**Fig. 7:**
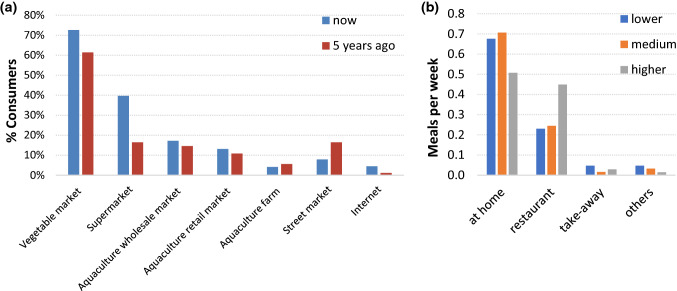
**a** Percentage of consumers purchasing seafood in different locations at the time of the survey and five years previously, **b** average weekly seafood meals consumed per person by location and social status. Restaurants include hotels and food stalls

Diverse consumption habits were supported by different product forms and choice of purchase location (Fig. 7a). Traditionally, consumers have bought seafood live from local “wet” markets, and this preference is seen most markedly amongst older consumers in our results. However, some supermarkets offer a wide range of purchase opportunities, including live fish and other seafood, kept in aquaria as well as more processed, frozen and value-added products. Younger and wealthier consumers, particularly from urban centres, were willing to buy more frozen or canned product from supermarkets or online which may be imported and of higher value. The shift to purchase of seafood in supermarkets and away from street markets is notable over five years. However, the data show that in general, the number of people who buy seafood from the majority of outlets have increased, reinforcing the growing consumption of seafood in Hubei. Wealthier consumers tended to eat out of the home much more often than consumers of lower social status (Fig. 7b), and this may reflect a choice towards higher value species too, which was also seen in urban areas where consumption of traditional species of grass and silver carp was slightly lower than in rural areas.

### Production survey

A total of 85 farmer production surveys were collected, including 77 carp farms (Table 5) and 8 crawfish, crab polyculture or shrimp farms. Four of the systems were based on pens in lakes, one was a rice/crawfish system in a paddy field and all others were pond systems, including three farmers who were growing lotus (*Nelumbo nucifera*) in one of their ponds. No lake/reservoir cage systems could be found to be still in operation, although the left-over equipment from derelict sites was found in several places.

**Table 5:** Carp farm production data, surveys collected by district and scale (Table 2)

	Wuhan	Jingzhou	Jingmen
Small	16	23	18
Medium	7	11	6
Large	2	2	0
Total	25	36	24

Carp polyculture was still the most commonly practiced form of aquaculture with on average 3.29 (± 1.48) different species cultured on each farm. Most commonly, grass carp, silver carp, bighead carp and crucian carp were cultured (Table 6), but farmers stated that species diversification was a strategy to combat lower farm-gate prices. On average farmers used 2.75 ponds for culture with 85% of respondents owning 3 or fewer with an average farm water area of 2.1 ha, discounting lake systems. Most commonly, farmers employed one carp polyculture grow-out pond with another pond for fingerlings, usually integrating grass carp with both silver carp and bighead carp, with around half including crucian carp. Farmers with three or more ponds sometimes rotated different species concurrently, sometimes growing lotus root as ponds became excessively eutrophic. Farmers with fewer ponds sometimes chose to grow lotus instead of fish in some years, alternating between different species according to the market. Most crawfish farmers produced no other species, although two farmers were also stocking silver carp and bighead carp.

**Table 6:** Number aquaculture facilities surveyed harvesting different species, from three districts of Hubei Province (*N* = 85)

District	Grass carp	Silver carp	Bighead carp	Crucian carp	Common carp	Black carp	Wuchang Bream	Crawfish	Other
Wuhan (*n* = 25)	20	19	19	7	5	1	1	0	3
Jingzhou (*n* = 37)	22	25	25	24	6	3	1	4	3
Jingmen (*n* = 24)	16	16	17	9	1	0	3	2	5

**Fig. 8:**
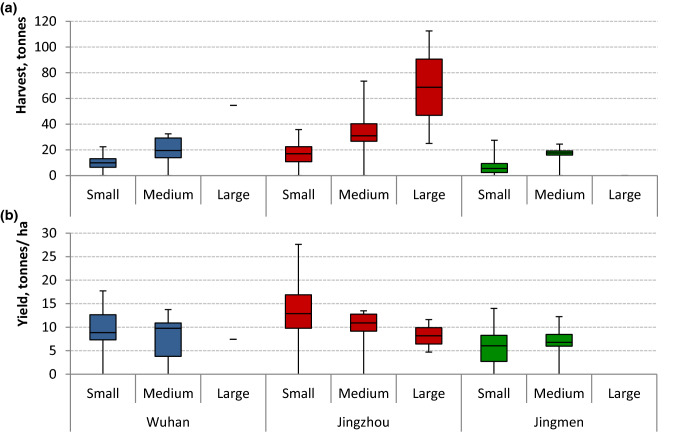
**a** Total harvest of fish (tonnes) from carp polyculture systems by farm scale, **b** yield per ha (tonnes) of fish harvested from carp polycultures in three districts of Hubei province by farm scale. Box and whiskers show minimum, quartiles and maximum (*N* = 77)

**Fig. 9:**
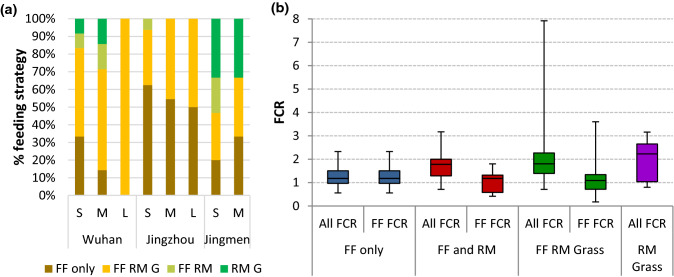
**a** Feeding strategies for carp polyculture in Hubei showing percentage of farmers using formulated feeds (FF), raw material (RM) and grass (G) supplementation, by scale; small (S), medium (M) or large (L). **b** Dry-weight adjusted feed conversion ratio (FCR) attributed to all feed materials (All FCR) and to formulated feed only (FF FCR) of carp polycultures fed formulated feed (FF), supplementary raw materials (RM) and grass in three districts of Hubei province. Box and whiskers show minimum, quartiles and maximum (*N* = 66)

The total farm harvest depended on both the scale and the area of production, with Jingzhou farms producing significantly more fish than in Jingmen (*p* = 0.002; Fig. 8a). Similarly, fish yields were also significantly higher in Jingzhou compare to Jingmen (*p* = 0.003), but overall, there was no difference between scales (Fig. 8b). However, there are clear differences and inconsistencies in production practices between different areas which make generalisation between scales impossible. Most evident is the difference between feeding practices in different areas (Fig. 9a).

While 90% of farms in Jingzhou districts used formulated feed, frequency of use was much lower in Jingmen (52%). In contrast, only 7% of farms in Jingzhou supplemented with unprocessed raw ingredients such as wheat or soybean compared to 50% of farms in Wuhan and 43% of farms in Jingmen. More than half (52%) of farms overall used rye grass to supplement feeding. Both raw material ingredients and grass were commonly grown on pond dykes and often fertilised using pond sludge, with some feed ingredients sourced from elsewhere. Use of grass was highest in Jingmen (62% farms) and lowest in Jinzhou (40% farms), whereas Jingzhou was much more likely to use only formulated feeds and Jingmen was more likely to use no formulated feed at all. Some differences could also be seen between scale, although this was not consistent between districts. Smaller farms seemed more likely to rely on formulated feed alone apart from in Jingmen.

It was found that the dry-weight adjusted FCR was significantly affected by the proportion of raw materials (*p* = 0.001) and grass in the diet (*p* = 0.015) but not by the scale or area of production. The FCR for formulated feed (i.e. not including the dry weight adjusted raw material or grass inclusion) was also significantly affected by inclusion of raw materials (*p* = 0.003) and grass (*p* = 0.011), suggesting that both supplements had an effect in improving the efficiency of formulated feed (Fig. 9b), i.e. although the overall FCR is increased, less expensive formulated feed is required to maintain performance. On average, the lowest FCRs related to formulated feed were seen when supplemented with raw ingredients and grass, which was commonly practiced in every district surveyed. There was no significant difference in the total harvested yields between the different feeding strategies, although the highest yield was with formulated feed only and the lowest yields were associated with no formulated feed.

## Discussion

### Consumption

The Chinese culture has a long tradition of seafood consumption encompassing a wide range of fish, crustaceans, molluscs, plants and other organisms. Fish are revered in Chinese culture as good omens of prosperity due to the similar pronunciation of the word fish (鱼,*yú*) and surplus (余, *yú*). This tradition gives fish high status within the national psyche for home consumption and particularly as a status symbol when eating out (Fabinyi 2011; Ning and Zhang 2020). As such, China is a pioneer of aquaculture dating back millennia (Weiman and Mengqing 2007). The status and demand for fresh, high-quality fish made it a priority for government food security programmes that drove the early stages of aquaculture expansion (Hishamunda and Subasinghe 2003; Cao et al. 2007; Sun and Collins 2013; Jiang 2017). While, in some Asian countries, aquaculture intensification has focused on more lucrative species for the export market (Little et al. 2018), the early intensification in Chinese aquaculture was largely to ensure its own domestic food security and for poverty alleviation (Hishamunda and Subasinghe 2003; Zhang et al. 2015; Jiang 2017). Estimated per capita seafood apparent consumption in China grew to over 40 kg per year (FAO 2018) by 2015 from less than 1 kg per year in 1949 (Cao and Sang 2019). The relatively low price of grass, silver and bighead carps were affordable for low-income families and provided the basis of aquaculture expansion and Chinese seafood consumption (Hishamunda and Subasinghe 2003). From the mid-1980s, the largest increase in carp species occurred, but the Chinese government considered that food security had been established and agricultural production became less subsistence oriented to a more economic focus (Li et al. 2017) including a higher focus on various export species, including shrimp (mainly *Penaeus vannamei*), tilapia (*Oreochromis spp*.) and more recently, red swamp crawfish (Yee 1999; Li et al. 2011; Zhang et al. 2017). However, as a large middle class has developed of around 0.5 billion people (Atsmon and Magni 2012; Wei et al. 2020), the more affluent, informed and technology savvy demographic are demanding a much richer variety of seafood (Crona et al. 2020). Diverse seafood demand is largely satisfied by domestic production, although demand for luxury imported species is also high (Weiman and Mengqing 2007; Fabinyi 2011) as the third largest importer of seafood at US$8.7Bn (Ortega et al. 2014; FAO 2018). The larger urban centres and richer East Coast have often been able to afford a richer variety of seafood products, but growth in seafood consumption in rural areas has been faster than in urban areas over the last few decades, and there is evidence to suggest that a larger proportion of society are beginning to access more diverse choices (Hishamunda and Subasinghe 2003).

The trajectory of diversification, described above, is supported by Hubei consumption data presented here, but as the survey is based on recall, it is only possible to ascertain whether or not people were consuming different species 5 years previously and not the total quantity consumed. However, data reveal that grass carp is still the most popularly consumed product but is less important, with purchasing power now allowing many sections of society to broaden their food choices to include a much more diverse menu of staple and status items alike. Even within the four domestic carp species, there are significant differences between price and consumer perception. Higher value species such as black carp and Wuchang bream are gaining popularity as higher status commodities compared to grass carp which has declined slightly in popularity (Fig. 6). Higher consumption of other high-value species, such as crawfish, swamp eel and others, can also be related to increased purchasing power (Chiu et al. 2013; Henriksson et al. 2018). High-value species are now much more highly consumed, even among medium to low-income respondents, including a 246% rise over 5 years in the number of people eating crawfish, for which production is rising considerably in Hubei province. Although exports of crawfish have increased, they are dwarfed by the rise in domestic consumption. While in 2003, 55% of the 45 thousand tonnes of Chinese crawfish production was exported, by 2017, less than 2% of the 1.23 million tonnes of production was exported (FAO 2019) with growth in the local market driving production. Although several observers have predicted Chinese seafood exports to weaken in the face of increased domestic consumption (Chiu et al. 2013), data show both imports and exports continue to increase (FAO 2020).

The data presented in Figs. 6 and 7 show people are also accessing a wider range of choice on how and where to consume seafood. People have traditionally purchased food from “wet” markets which offer consumers assurance and trust over the perceived freshness of the goods that they are purchasing, i.e. that it is “immediate” and not preserved in any way. Consumers may still buy food on a daily basis to ensure freshness with much less reliance on home refrigeration (Zhong et al. 2019). Fish is especially associated with freshness, the Chinese character for the word “fresh”, xiān (鲜) being a conjunction of the characters for fish (鱼) and sheep (yáng, 羊). In many cases, especially among older demographic, Chinese demand a degree of freshness that can only be provided by purchasing live fish, hence the presence of aquaria in many restaurants, supermarkets and wet markets. Fish are transported in oxygenated tanks on lorries, which add costs compared to transporting processed products. The penchant for fresh seafood also adds risk, in that aquaculture production management must track demand more closely as there is little capacity for long-term storage (Li et al. 2011). There is a shift from people buying solely from wet markets to a wide range of purchasing options meeting the demand for freshness in many cases, but also convenience. The status of wet markets has recently been complicated by COVID-19, which is suspected to have originated in a wet market in Wuhan, and there is not only increasing domestic and international scrutiny over their public health impact (Nature Food 2020). The term “wet market” encompasses a large range of different outlets where consumers can buy fresh produce and it remains to be seen what effect the recent pandemic will have. Purchasing options not only include other forms of wet markets such as vegetable markets, but also include a wealthier demographic, frequently eating out and a small percentage of younger, technology focused population willing to purchase food via the internet and other high-tech vending options. Consequently, Chinese consumers are spending a higher proportion on seafood and on higher trophic and luxury species (Fabinyi 2011). High-tech purchasing options are revolutionising choices and how some consumers choose to interact with their food, reflecting the development of a modern, more affluent, technology-integrated Chinese society.

### Production

Production data supported the consumption trends overall, but aquaculture production data in Hubei province from China’s MOA and FAO statistics at the time (FAO 2016; MOA 2016) suggested a somewhat conflicting situation regarding carp farming compared to our consumption and production data. However, our findings were later confirmed by the more up-to-date provincial data (MOA 2019) that showed declines in low value carp production, in favour of crawfish and higher value fin-fish including black carp and Wuchang bream. Our findings for Hubei were in agreement with those of Chiu et al. (2013) who found similar diversification in Jiashan and Qiandaohu.

Evidence from key stakeholders suggested that the financial margins in carp farming have become much tighter with wholesale prices at Wuhan market falling and input costs rising. However, where Chiu et al. (2013) found that farmers were absorbing the extra costs, our survey found that farmers were changing practices to maintain margins. One feed producer declared that their sales of carp feed had fallen by as much as 30% in recent years. Belton et al. (2020) argued that there was a pathway to commoditisation of seafood through intensification from niche products to consolidated mass production. The common perception is that farmers are moving towards more intensified systems based on use of formulated feeds; however, while this had been the case historically, farmers were now finding it more profitable to adopt a less intensive model. Many farmers were reducing formulated feed input and supplementing with grass, soybean and other raw feed ingredients grown on the pond bank, with or without fertilisation from pond sludge. The FCRs presented in Fig. 9 show that the inclusion of raw materials and grass can improve the FCR of the formulated feed although the range of FCRs is more varied within this group. Feed is usually the most expensive operational cost of an aquaculture enterprise at between 50% and 70%, followed by labour and energy/fuel (Liu et al. 2017). Therefore, there is a trade-off between the extra labour needed to produce, harvest and sometimes process crops on the dykes compared to the cost of formulated feed. However, as most of the farms are family enterprises, the labour is likely to be flexible and shared within households. The reversion to more traditional methods questions the notion of sustainable intensification, as the process of intensification through formulated feed use alone for carp-based aquaculture in China has clearly not been economically sustainable. This may be for several reasons. The overarching reason is the resilience of the industry in responding to challenges and how farmers perceive risk. Out of all the farmers interviewed, 10% made a loss and 40% made less than 20 thousand CNY, compared to an average income of 33.6 thousand CNY for a Chinese agricultural worker in 2016 (National Bureau of Statistics of China 2017). As profit margins reduce, a common response is to become more competitive through better efficiency (to produce more with less), although de-intensification is also regarded as a risk mitigation strategy (Little et al. 2018). Li et al. (2011) have suggested that the potential for further intensification within a small-scale producer-dominated carp sector is constrained because of limited capacity for additional technological innovation and the low value of the species. Recent innovations such as the Intensive Pond Aquaculture (IPA) system, described as an in-pond raceway that allows better waste management (Cremer et al. 2014), have shown promise, particularly to improve the efficiency of formulated feed use, but investment costs remain high and market share is unreported. If these systems become commercially established, it is unlikely to be by current small-scale pond operators. Coupled with increased uncertainty regarding risk, including market factors, rapid change in demographics, urbanisation, industrialisation, pollution and other environmental pressures, including climate change (Li et al. 2016), intensification may not be the best solution to counter falling profitability of small-scale aquaculture.

De-intensification was described by Little et al. (2018) as a risk mitigation strategy for high-value species such as shrimp or prawns where low yields could still return a profit. However, this is not the case with Chinese carp which is typically low to medium value, and the results showed that smaller scale farms were more likely to use formulated feed only and have higher yields per hectare. This is most likely due to the proportionately larger labour effort required to use crop diking techniques in small-scale farms that must be fitted around other economic activities. The situation regarding Chinese labour is complex, but the basic household unit still dominates agricultural production, with family members adopting diverse income supplementation strategies (Chen and Zhao 2017). The small household farm unit rarely provides sufficient income, and members are often compelled to find additional or alternative income, locally or further afield (Chen and Zhao 2017). Although, in recent times, rural populations have been attracted to urban centres for employment opportunities, there are state disincentives to migration from agricultural centres, associated with access to state benefits and relinquishing of rural land lease rights, resulting in decreasing outward migration from rural centres and a larger local labour pool (Chen and Zhao 2017). However, particularly young male household members often move to find work, splitting families and leaving female and elderly members to provide rural labour (Doczi et al. 2014; Chen and Zhao 2017; Li and He 2020). Small-scale farms may, therefore, have labour reduction through migration and unable to pay for recruitment, whereas larger farms may be able to afford to tap into local labour availability (Li and He 2020). Li and He (2020) found similar trade-offs in rice culture, but Sharma et al. (1999) found that smaller scale farms were generally more efficient and profitable. The pattern may also be connected to the small-scale producer structure of Chinese agriculture where there is a high tendency for top-down control and producers tend to adopt similar practices to each other in any particular area (Sun and Collins 2013; Li et al. 2017).

Other changes along the pathway to full commoditisation reported by Belton et al. (2020) include structural innovation by supply chain transformation. While there has been upstream supply chain transformation allowing for intensification, entrenched traditions regarding the purchase and consumption of seafood have not significantly transformed the supply of seafood from the farm to the consumer, which have prevented further commoditisation through processing, and hence, value addition demonstrated in, e.g. the salmon value chain (Newton et al. 2014; Stevens et al. 2018). Instead of post-harvest supply chain transformation, data suggest that in the face of stagnating prices for traditional species, successful risk mitigation strategies of Chinese inland aquaculture were to diversify species production to better match variable consumer demand. Silver carp were especially low in value. Whereas silver carp made up the majority of Chinese carp production for human consumption prior to the 1980s (FAO 2020), they were often used as a direct feed input for more highly valued species in Hubei at the time of the survey. Adult silver carp were being minced for swamp eel (*Monopterus albus*) feed or the fry as live feed for Mandarin fish (*Siniperca chautsi*). Prices are more likely to fluctuate for any particular species, according to short-term demands of consumers buying fresh produce on a daily basis. However, while market information in the past was often based on word of mouth and middlemen networks (Chiu et al. 2013), farmers in Low- and Medium-Income Countries generally are now much more empowered to make decisions through better information and telecommunication technology (El Bilali and Allahyari 2018). In many cases, farmers with one or two ponds were either moving away from carp production entirely towards more lucrative species such as crawfish (*Procambarus clarkii*) or produced different species on a year-to-year basis such as crucian carp (*Carassius carassius)* or black carp (*Mylopharyngodon piceus*), depending on market conditions. In some cases, farmers with three or more ponds were employing a concurrent rotation model between carps and other species including plants such as lotus root cycled year-to-year to broaden their prospects and make better use of their nutrient inputs. Lotus root had the added advantage that it is less labour intensive than fish culture (Edwards 2012). Diversification to more lucrative species is not new as farmers seek more profit (Weiman and Mengqing 2007), but single species production is still exposed to risk from market shocks, and small-scale producers are especially prone to the risk associated with installing expensive technology required to manage some high-value species (Yee 1999). A compromise between intensive production and more diversified traditional production lends itself to a rotation model where a range of products can be produced per cycle and the risk of relying on fewer species can be reduced.

Carps have often being regarded as relatively benign in their environmental impact and their demand on resources compared even to other cultured finfish (Naylor et al. 2000; Chiu et al. 2013; Jia et al. 2013; Roberts et al. 2015), although in some places, their introduction has cause concern for local ecosystems (e.g. Irons et al. 2007). However, their sheer production volume in China means that any changes in their production techniques can have considerable local and global environmental trade-offs in globalised supply chains (Li et al. 2011; Cao et al. 2015; Henriksson et al. 2015; Newton and Little 2018).

Particularly The changing demand of Chinese aquaculture on feed resources is highlighted in this research. Concerns over feed consumption in aquaculture are especially linked to the growing proportion of marine ingredients that aquaculture consumes (Tacon and Metian 2015). Currently it is estimated that China consumes approximately 30% of global fishmeal supplies to be used mainly in aquaculture (Cao et al. 2015). Estimates for fishmeal inclusion are on average around 3% to 3.5% in carp diets (Weiman and Mengqing 2007; Cao et al. 2015), which together with data from Tacon and Metian (2015) equate to around 0.5 million tonnes of fishmeal (roughly 10% of global supply). Although some commercial data suggest that this is likely to be an over-estimate, the proportion of global fishmeal that is taken up by Chinese carp culture is significant. The Chinese fishmeal industry itself has received significant criticism for being indiscriminate and unmanaged, contributing to considerable economic damage to regional fisheries (Cao et al. 2007, 2015; Chiu et al. 2013; Zhang et al. 2019), not only in Chinese waters but also further afield, such as in African fisheries (Hicks et al. 2019; Pauly 2019) as Chinese fisheries become critically overfished (Zhang et al. 2019). Growing pressure on supplies has led to increasing dependence on soybean imports, from the USA and South America, for use in all livestock production, increasing food insecurity by dependence on external markets and contributing to more widespread global environmental degradation (Roberts et al. 2015; Newton and Little 2018; Malcorps et al. 2019). There is no doubt that intensification has allowed for rapid rises in production; however, global pressure on commodities to supply increasing feed demands has been blamed by some for increasing the cost of fish production, while increased production has resulted in stagnation of farm-gate prices. This is perceived to cause loss of profits (Li et al. 2011) and is threatening the long-term sustainable growth of carp production in China as farmers seek more profitable alternatives. However, as diversification to higher value species increases, the dependency on higher grade feed inputs and associated reliance on raw materials such as marine ingredients may also increase (Chiu et al. 2013). There is a huge untapped potential for circular economy solutions to China’s feed ingredients shortage if seafood was more processed, separated, and consumed more efficiently (Cao et al. 2015; Jackson and Newton 2016; Wang et al. 2017c; Stevens et al. 2018). However, a cultural shift in consumption patterns from live fish to processed and preserved seafood would be needed to allow for the necessary supply chain transformation, which is unlikely in the short to medium term.

#### Regulation

China’s top-down system is very powerful, which can bring changes to the industry very quickly, for good or bad (Cao et al. 2017). In the case of aquaculture, the complete removal of cages and pen culture systems from lakes, reservoirs and rivers/ditches, was effectively and swiftly implemented (2016–2017) aiming to protect the local environment for multiple stakeholders but simultaneously removing people’s livelihoods with little compensation and no alternative (Wang et al. 2017a). Under the 12th five year plan (2011–2015), China adopted a much more environmentally conscious approach to its development (Doczi et al. 2014) including the “Three Red Lines” policy designed to control water withdrawal, utilisation efficiency and water quality (Doczi et al. 2014; Wang et al. 2017b). Wang et al. (2018) provide a list of regulations that have been introduced to Hubei since 2002 that have curtailed freshwater aquaculture expansion. Key to this list is regulations dating from 2011 to 2015 that have banned the use of feeds and fertilisers in lakes and reservoirs, prevented the modifications to lakes and reservoirs by using pens or dykes to enclose areas for culture, and culminated in bans of aquaculture in lake and rivers (The standing committee of the people’s congress of Hubei province 2012, 2014; State Council of China 2015; The standing committee of the people’s congress of Shandong Province 2018). Despite large resources and efforts to optimise efficiency, China has suffered from water scarcity and degradation in recent years (Doczi et al. 2014; Wang et al. 2017b). As China’s economy developed, it prioritised urbanisation and industrial manufacturing over agricultural production in the Yangtze (where Hubei is located) and Pearl River basins, compounding the competition and pollution issues between different stakeholders (Li et al. 2011, 2017; Doczi et al. 2014). Such competition, degradation and scarcity have resulted in reduced grain harvests (Wang et al. 2017b) and economic damage (Doczi et al. 2014). Water scarcity and the associated pressures are expected to increase with climate change (Wang et al. 2017b). Subsequently, the government imposed regulations to improve water quality, increase the availability of drinking water and ultimately to safeguard water resources for continued economic growth (Doczi et al. 2014; Wang et al. 2017b). At the time of the survey, in some peri-urban areas, ponds were being filled in for other uses such as recreation or urban expansion to support this transition. Ponds built into lakes using dykes and other ponds in urban areas were being either returned to a “natural” lake status or simply being filled in, especially in areas where other uses of land and water were being prioritised (Fig. 5). Stakeholders declared that environmental restrictions were becoming tighter as water resources are no longer free to everyone, as competition between human consumption, urbanisation, agricultural and industrial uses become more apparent and water shortages become more prevalent (Doczi et al. 2014; Wang et al. 2015). As aquaculture facilities have often been close to expanding urban areas, the lakes and reservoirs are becoming increasingly important for drinking water and other uses (Wang et al. 2017a). Environmental pollution issues from aquaculture and urbanisation have led to toxic algal blooms, and together with other pollution such as chemical treatments from aquaculture and industrial uses have compromised the safety of these shared water bodies especially as a source of drinking water but even utilisation for industry (Cao et al. 2007; Li et al. 2011; Jia et al. 2013; Zhang et al. 2015; Wang et al. 2017a, 2018). Unfortunately, the diversification away from formulated feed may exacerbate eutrophication, because there tends to be higher direct and indirect wastage from farm made feeds due to their poorer integrity and inferior nutritional balance, respectively (Weiman and Mengqing 2007). Although cage culture on reservoirs and lakes is now illegal (Wang et al. 2018), filter feeding species such as silver carp or bighead carp are sometimes used to remediate eutrophied lakes (Jia et al. 2013). Some efforts have been made to mitigate the economic shocks of reduced production from lakes and reservoirs by allowing low intensification pond aquaculture in the surrounding wetlands which had previously been converted for more intensive culture (Wang et al. 2018). These include rice-fish and indoor systems, but their scale has been insufficient to compensate for the large loss in available area once provided by lakes and reservoirs. As urbanisation and environmental protection legislation continue to take force, it is likely that more farmers will be displaced and culture area reduced. Time will tell what effects this has on overall aquaculture in China and shifts in consumption patterns.

## Conclusions

The unparalleled rise in production of aquaculture, together with other agriculture changes, in China through the twentieth century has been a massive success story that has brought food security to a poor and under-nourished population. Seafood has a special place in Chinese nutrition as a status symbol leading to high demand for a diverse menu of quality, fresh aquaculture produce which places pressures on supply chains to coordinate supply with demand. The combination of changing demographics and regulation have placed pressures on farmers to meet demand and stay profitable. In spite of a trajectory of intensification since the mid-1980s supported by a large and competitive formulated feed sector, farmers appear to have diversified their production models and species to maintain margins but also respond to market opportunities. On the one hand, they have responded to meet demand for a diverse selection of seafood, by increasing production of a range of higher value species employing integrated and rotation systems. They have also reduced costs by strategic substitution of formulated feed using labour intense farm-mixed feeds and using raw ingredients grown on the pond dykes. Given the demographic trends in China this approach may not be sustainable.

As urbanisation has increased and government has focused on industrialisation, peri-urban fish culture has often had to make way including measures to reduce pollution in shared water sources, particularly those supplying drinking water to larger urbanised centres. Hubei may not be unique in this respect as urbanisation and industrialisation increase in the major river valleys of Central and Eastern China. China will continue to encounter issues of raw material supply and difficulties in meeting fluctuating demand. This could perhaps be partly solved by moving away from the heavily engrained preference for non-processed, fresh produce to more processed and preserved products and improving the efficiency of marine ingredient inclusion in some feeds.

## Supplementary Information

Below is the link to the electronic supplementary material.

Supplementary file1 (PDF 905 KB)
