# Supplementary material for: Intensification, regulation and diversification: The changing face of inland aquaculture in China
Source: Ambio. 2021 Mar 5;50(9):1739–56. doi: 10.1007/s13280-021-01503-3 (PMC7935007; doi:10.1007/s13280-021-01503-3)
Supplement: Supplementary file 1 — Supplementary file1 (PDF 905 KB) [file 13280_2021_1503_MOESM1_ESM.pdf]

Ambio Electronic Supplementary Material
This supplementary material has not been peer reviewed.

Title: Intensification, regulation and diversification; the changing face of inland aquaculture in China

Consumption and production surveys conducted in Hubei province

Authors: Richard Newton, Wenbo Zhang, Zhaoxing Xian, Bruce McAdam, David C Little

Consumption survey (English)

Research question:
The consumption status and consumption trends of aquatic products during the rapidly development in recent years.

1) **Current consumption status and feature.**
2) **Consumption trend: the changes of consumption species, consumption habit, purchasing place and consumption location.**
3) **How consumption influences production.**

1. Consumption structure related to species, family structure, age-group, and the education degree.
2. The proportion of aquatic food expenditure in total food expenditure.
3. The carp consumption in rural areas compare with that in urban areas. (wondering that are city people moving away from eating carp and tend to eat more non-carp fish?)
4. The consumption ratio of captured fish and cultured fish. (also compared consumption of freshwater fish with saltwater fish)
5. Do people increase interest in higher value carps and other fish? （趋势）
6. Are wealth people more frequent to consume captured fish?
7. Is there any certificated aquatic products sale in markets? Do consumers prefer to buy certificated products even if more expensive?
8. The position of buying aquatic species. (supermarket or traditional wet fish market), the reason they prefer to buy fish in this place (cleanliness of the place, good hygiene of the supermarket? Or cheaper price of the local market?)

Consumption survey

District code：1）Wuhan 2）Jingzhou 3）Jingmen

A. Respondent's general informant:

1. Gender: 1）male 2）female

2. Where do you live: 1）urban 2）rural

3. Age:

   (1).20 以下， (2)20-29， (3)30-39， (4)40-49， (5)50 以上

4. How many family members live with you? ___________

5. Education status:

1）under secondary school 2 ）high school 3）undergraduate 4.）master and above

6. Do you have children under 16 years old?

1） Yes 2）No

7. Are you the major buyer in your family?
   Yes No

8. Your current working situation:

   1）Has a full time job in company or organization；2）Has a part time job in company or organization; 3）Self-employed or starting a business yourself；4）Employment in retirement；5）Retirement；6）Student；7）Unemployed；

9. Please make a choice below which matching your annual income. (Yuan)

   1）0-40000；2）40001-80000；3）80001-120000；4）12001-160000；5）160001-200000；6）above 200000.

10. The average annual income of your family (per capita) ■ around_____________Yuan.

B. The purchasing place of aquatic products.

1. Where are you usually buy aquatic food?
   1. Vegetable market 2. supermarket 3. Aquaculture wholesale market 4. Aquaculture retail market 5. Aquaculture farm 6. street market 7. Internet

2. Please choose the reasons why you are willing to purchase aquatic foods here: (multiple-choice?)

   1）convenience, cleanness and more hygiene

   2）it demonstrates my social status

   3）can buy high quality aquatic products here

   4）there are good food safety guarantee here.

   5）aquatic food are cheaper here.

   6）close to home.

3. Have you changed your aquatic product's purchasing place?
   Yes No
4. If your purchasing place changes, what's your previous purchasing place?

1. Vegetable market 2. supermarket 3. Aquaculture wholesale market 4. Aquaculture retail market 5. Aquaculture farm 6. street market 7. Internet

5. The reason of changing purchasing place:

6. If you buy aquatic foods in supermarket now, do you intend to buy aquatic foods online in the future?
   Yes   No

7. Can you distinguish cultured fish and captured fish?
   Yes   No

8. What kind of fish you prefer to buy?

1) Cultured species 2) captured species 3) it doesn't matter.

9. The reason of why you are wiling or not willing to buy captured fish:

10. Total food expenditure of family:
    Please estimate your family food expenditure monthly is ________Yuan; and the aquatic products expenditure accounts for________%.

C. consumption pattern of aquatic products

1. Animal-protein consumption in 7 preceding days

	fish	crustacean	shellfish	Egg	Milk	pork meat	Mutton meat	beef meat
How many days								

2. how many aquatic foods you have eaten ever, please answerr the question according to the instruction?

Please write down your consuming frequency in preceding 7 days, for example: 3 times, 4times

Form of aquatic product:

1) Live (2) chilled (3) unjudgeable (4) smoked (5) others

Consuming location:

1）At home； 2）Restaurant/hotel/food stall；3）Take away；4）Others

Purchasing place:

1) Vegetable market 2. supermarket 3. Aquaculture wholesale market 4. Aquaculture retail market 5. Aquaculture farm 6. street market 7. Internet

	grass carp	silver carp	bighead carp	black carp	crucian carp	common carp	bream	Catfish	long snout	bass	wuchang	eel	shrimp	crab	molluscs	crayfish

									catfish		beam					
consumption frequency																
form of aquatic food																
purchasing place																

3. Which aquatic product you most most frequently consumed 5 years ago? （multiple-choice?）

grass carp	silver carp	bighead carp	black carp	crucian carp	common carp	bream	Catfish	longsnout catfish	bass	wuchang beam	eel	shrimp	crab	molluscs	crayfish

4. How many times you eat in the restaurant or hotel per month in general?
   1) Under 5 times 2) 5-10 times 3)11-15 times 4) above 15 times

5. How many kinds of aquatic foods you will consume when you in the restaurant?
   （1）0 （2）1 type of aquatic food（3）2 types of aquatic foods（4）3 types of aquatic foods（(5）above 4 types of aquatic foods.

D. Cognitive degree of Quality Certification of Aquatic Product:

1. Have you heard of Quality Certification of Aquatic Product? (such as: green aquatic product，free-pollutant seafood，organic seafood)

   1)Yes, I heard of it 2) No, I haven't heard of it

2. What kind of aquatic product belongs to category of Quality Certification of Aquatic Product?
   1) green color 2) safety and pass the quality inspection of national standard 3) benefit to health care 4) unclear
3. Have you ever bought Quality Certification of Aquatic Product?

   1) always 2) regular consumption 3) occasionally 4）never

4. where are you usually buy Quality Certification of Aquatic Product?
   1）supermarket 2）vegetable market 3）aquaculture wholesale market 4）online
5. why are you willing (or not willing) to buy Quality Certification of Aquatic Product?

6. how many extra fee you are willing to pay for the Quality Certification of Aquatic Product?
   1)below 10% 2)20%-30% 3)31%-40% 4)41%-50% 5) above 50%

Consumption survey (Mandarin)

Research question:

The consumption status and consumption trends of aquatic products during the rapidly development in recent years.

比较农村与城市之间消费现状及特征的差异

特征：水产品在日常蛋白食品中的比重，消费偏好

消费趋势：看消费习惯是否有改变，消费品种是否有改变，出去消费的次数，购买的水产品的场所

消费时如何影响生产的。

Research questions related to consumption surveys:

1. Consumption structure related to species, family structure, age-group, and the education degree.
2. The proportion of aquatic food expenditure in total food expenditure.
3. The carp consumption in rural areas compare with that in urban areas. (wondering that are city people moving away from eating carp and tend to eat more non-carp fish?)
4. The consumption ratio of captured fish and cultured fish. (also compared consumption of freshwater fish with saltwater fish)
5. Do people increase interest in higher value carps and other fish? （趋势）
6. Are wealth people more frequent to consume captured fish?
7. Is there any certificated aquatic products sale in markets? Do consumers prefer to buy certificated products even if more expensive?
8. The position of buying aquatic species. (supermarket or traditional wet fish market), the reason they prefer to buy fish in this place (cleanliness of the place, good hygiene of the supermarket? Or cheaper price of the local market?)

城市编号：1）武汉 2）荆州 3）荆门

第一部分：个人基本信息：

1. 您的性别：1）男 2）女

2. 户口所属地：1）城市 2）农村

3. 您的年龄

   (1).20 以下，(2)20-29，(3)30-39，(4)40-49，(5)50 以上

4. 在本市一起居住和生活的家人有__________人

5. 您的教育程度是：

1）初中以下 2 ）高中 / 中专 3）大学 4.）硕士及以上

6. 有无 16 岁以下的孩子：

1） 有 2）无

7. 是否家庭的主要购买者？

是 否

8. 您目前的就业情况：

1）在单位/公司有固定工作；2）在单位/公司有一份临时工作；3）个体户/自谋职业/自己创业；

4）退休后返聘；5）退休在家；6）还在上学；7）一直无业/待业；

9. 请选择最符合您 **[年收入]** 状况的选项

1）0-40000（4 万元）；2）40001-80000（8 万元）；3）80001-120000（12 万元）；

4）12001-160000（16 万元）；5）160001-200000（20 万元）；6）20 万元以上。

10. 家庭**[人均年收入]**呢？ ■ 大约为____________万元。

11. 家庭食物支出方面：

请您估算一下，您全家一个月家庭的食物支出大概是____________元；其中，水产品大约占____________%。

12. 调查水产品对不同收入的人群消费的重要性：

一个星期内吃过的含有蛋白质食物（Animal-protein consumption in 7 preceding days）

	鱼	虾蟹	贝类	鸡蛋	牛奶	猪肉	羊肉	牛肉
有几天是吃过这种食物								
估计一下一共消费的量（kg）								

第二部分：水产品的消费模式

1. 请问下表所示的水产品当中，您都吃过哪些？请安提示填写下表：

消费频率请填写你一周内吃过该品质的次数，如 3 次，4 次

食材形式的可选择项是：

1）冻品； 2）冰鲜品；3 腌制品；4）干制品 5）罐制品；6）无法判断

消费场所的可选项是：

1）在家； 2）在饭店/餐馆/排挡；3）外卖；4）其他

(Frequency of fish consumption in the preceding 7 days by type)

	草鱼	白鲢	鳙鱼	青鱼	鲫鱼	鲤鱼	鳊鲂	鲶鱼	鮰鱼	鲈鱼	武昌鱼	黄鳝	虾	蟹	贝类	小龙虾
七天内吃过的次数																
食材形式																
消费场所																

2. 目前消费的主要品种和之前（五年前）消费的主要品种是否一样？

1）是　　2）否

3. 如果现在消费的主要水产品和以前（五年前）的不一样，以前主要消费的水产品是什么种类：（多选）

草鱼	白鲢	鳙鱼	青鱼	鲫鱼	鲤鱼	鳊鲂	鲶鱼	鮰鱼	鲈鱼	武昌鱼	黄鳝	虾	蟹	贝类	小龙虾

4. 为什么主要的消费品种会发生改变？

____

5. 五年前您主要购买的是那种水产品形式：

1）鲜活品； 2）冰冻品；3）腌制品；4）干制品； 5）罐制品； 6）无法判断

6. 一个月有多少次在餐馆或酒店吃饭？

（1）5 次以下 （2）5-10 次（3）11-15 次（4）15 次以上

7. 每次在餐馆吃饭时都会消费多少种水产品？

（1）0 种 （2）1 种（3）2 种（4）3 种（5）4 种以上

第三部分：水产品购买场所

1. 目前购买水产品的主要地方：

买水产品的地方：1.菜市场，2.大型超市，3. 海鲜批发场，4.海鲜零售市场 5. 养殖场，6. 路边摊 7. 网络

2. 购买水产品的主要场所有发生过变化吗？

有 没有

3. 假如有发生变化，之前的主要购买场所是：

1. 菜市场，2. 大型超市，3. 海鲜批发场，4. 海鲜零售市场 5. 养殖场，6. 路边摊 7. 网络

4. 请选择您改变购买水产品场所的原因：（多选？）

1）方便，而且干净卫生

2）更能体现我的身份地位

3）能购买到品质更好的水产品

4）购买的水产品食品安全更有保障

5）价格更加便宜

6）距离家里比较近

5. 以后会不会选择在超市购买水产品？

会　　不会

6. 以后会不会尝试在网上购买水产品？

会　　不会

7. 是否有特意倾向去选择购买野生水产品？

是　　否　　无所谓

8. 是否能区分养殖水产品和野生捕捞的水产品

能　　否

9. 愿意或不愿意购买野生水产品的原因

____

10. 是否有特意倾向去选择购买海水水产品？

是　　否　　无所谓

11. 是否能区分淡水水产品和海水水产品

能　　否

12. 愿意或不愿意购买海水水产品的原因

____

第四部分：有质量认证水产品的认知程度：

1. 是否听说过有质量认证的水产品？（如无公害水产品，绿色水产品，有机水产品）

听说过， 没听说过

2. 什么样的水产品才算是有质量认证的水产品？

（1） 绿色的（2）安全的，经过国家质量检查认证的（3）保健的（4）不清楚

3. 是否购买过带有质量认证的水产品？

（1） 经常购买 （2）偶尔 （3）购买过 （4）从未购买过

4. 通常在哪儿购买带有质量水产品？

1）超市 2）菜市场 3）水产品批发市场 4）网上

5. 愿意或不愿意购买带有质量认证水产品的原因

6. 愿意多付百分之几的钱买有质量认证的水产品？

（1）10%以下 （2）20%-30% （3）40% - 50%（4）50%以上

Production Survey

除非另外注明，所有问题都是关于去年全年的，并且针对正在访问的养殖场
777=不确定/不愿回答　888=不知道　999=不适用

Section 1 Survey and Interview details（***调查之后归类**）

Table 1.1 关于调查

**SurveyCD** 养殖场编号	***PrimaryspeciesCD** 主要的养殖对象	***FarmSystemCD** 养殖模式(系统)	***FarmScaleCD** 养殖规模
- - - - -			

Table 1.2 关于采访

**Survey Forms**			**ACCESS Database**		
**Date collected** 数据收集日期	**Collected by CD** 收集人	**SurveyCheckByCD** 问卷审核人	**Date entered** 数据输入日期	**Entered by CD** 输入人	**Checked by CD** 数据审核人

Table 1.3 养殖场地理位置

**Province** 省	**District** 地区	**SubDistrict** 县	**Village** 行政村	自然村/大队	**GPS Coordinates**GPS 调研发生地点坐标					
					**East** 东经			**North** 北纬		
							.			.

Table 1.4 受访对象、管理人员（场长、经理）和养殖场拥有者（老板）的信息

**Farm RoleCD** 其在养殖厂角色	
**Owner RelationCD** 和拥有者（老板）关系	
**Full Name** 姓名	
**GenderCD** 性别	
**Age** 年龄	
**EducationCD** 学历	
**Home Km From Farm** 家和养殖场的距离	
**Tel.Number** 电话号码	
**YearBeganAquaculture** 哪年开始水产养殖	
**PreviousOccupationCD** 之前的职业	

Section 2. Sustainability Perceptions

Table 2.1 你认为哪些因素将会对今后几年的养殖有积极影响或消极影响？

	**SustFactor** 详细因素	**OverallRank** 总排序	**Response** 应对措施
**Negative** 消极因素			
**Positive** 积极因素			
**Uncertain** 不确定			

Section 3. Household

Table 3．1 Household Income 家庭收入

	**IncomeSourceCD** 收入来源	**% Last YrsNet Income** 占收入百分比
Last Year 去年一年	水产养殖	

Table 3.2 去年从水产养殖获取的净利润或者净亏损是多少？

**To Respondents Household**<br>**针对访问对象的家庭（户）**	**Owner (if not respondent & known)**<br>**养殖场拥有者**

Table 3.3.主要贷款

**Any loans?** 有无贷款？	
**What for?** 贷款用途	
**CreditTermsCD** 赊欠的协议	
**CreditInterest %**赊欠的利息	

Section 4. Farm details

Table 4.1 详细信息（仅指正在调查的养殖场）

**Farm Trading Name** 养殖场商业用名	
**TraceabilityRegistrationCD** 质量认证类型无公害绿色有机	
**YearFarmEstablished** 养殖场哪一年建成	
**PriorLandUseCD** 之前土地使用情况	

Section 5. Aquaculture land holdings

*Aquaculture land-uses only 仅水产养殖用地

**CurrentUseCD** 当前用途	
**OwnershipCD** 产权关系	
PondCost 塘租（每年每亩租金）	
**ContractLengthYrs** 合同年限	
**MangementCD** 管理方式	
**Full-time staff** 全职的工人数量	
**Land Area** 土地面积	
***TotalWaterArea** 总的 水域面积	
**AreaUnitsCD** 面积单位	
**No. of Ponds** 池塘数目	
**No of other sites** 同一家其他养殖场数目	
**Total areas in other sites** 其他养殖场总面积	
**Total production in other sites** 其他养殖场总产量	

Section 6 – Infrastructure& Production for the visited farm

Table 6.1 养殖场基础设施

ContainmentUse CD 养殖水域用途	**Containment System CD** 养殖水域类型	**No units** 数目	**Total Water Area** 总的水面积	**Area Units CD** 面积单位	**Max water Depth (m)**最大水深（米）	**Fully/partially lining** 全部/部分覆盖物	**PondLining material CD** 池塘衬砌物（水泥，塑料膜）

Table 6.2 平常做什么养殖记录吗

**RecordCD** 记录内容	**Yes (tick)有（标记）**
Feed 饲料	
Growth 生长率	

Water quality 水质	
Chemical use 化学品使用	

Table 6.3 生产-仅指被访养殖场在去年全年的产量

**Species CD** 养殖放养品种								
	**Production Scheduling** 生产调度							
	平均养成时间<br>(放苗到收获)	Days[ ]<br>month[ ]						
	每年的批次							
	**过去 5 年总的批次(含正在进行的)**							
**Adult** 成鱼养殖	**SeedSourceCD** 苗种来源							
	**Select**	苗种均重（克）						
		苗种均长(cm)						
	平均苗种成本							
	**CostUnitCD** 成本单位							
	养殖密度 No/m^2[ ] m^3[ ]							
	到收获时的成活率%							
**Harvest**	**Select**	收获时的平均体重(kg)						
		平均头数/kg (虾/蟹)						
	上一次捕捞收获总量（吨）							
	去年收获总量（吨）							
**Marketing**	如何销售?							
	销售到哪里?							
	**Avg Price/kg** 平均价格/千克							
	**Min Price/kg** 最低价格/千克							
	**Max Price/kg** 最高价格/千克							
	*距市场的距离（公里）							
	*到市场的时间（小时）							

Table 6.4 苗种培养和分阶段养殖—仅针对鲤科鱼类

**FRY** 鱼苗培育	**SeedSourceCD** 苗种来源							
	**Select**	苗种均重（克）						
		苗种均长(cm)						
	平均苗种成本							
	**CostUnitCD** 成本单位							
	养殖密度 No/m^2[ ] m^3[ ]							
	到收获时的成活率%							
**Fingerling** 鱼种	**SeedSourceCD** 苗种来源							
	**Select**	苗种均重（克）						
		苗种均长(cm)						
	平均苗种成本							
	**CostUnitCD** 成本单位							

	养殖密度 No/m^2[ ] m^3[ ]					
	到收获时的成活率%					

Table 6.4 其它野生的（非投苗）种类？

Non-stocked species 野生种类				
Total production 总产量				
Production Units CD 产量单位（填重量单位）				
Non-stockedUseCD 收获（捕出）后去向				
TotalValue 总的价值				

Table 6.5 在上个养殖周期中立体农业（种植业）的收获

**AgriSpecies CD** 种植业种类	**Area cultivated** 面积	**Area Units** 面积单位	**Production useCD** 产品去向	**TotalValue** 总的价值

Table 6.6 养殖场的动物

**AnimalCD** 动物种类	**Count of Animals** 动物数量	**Production use** 产品去向	**TotalValue** 总的价值

Section 7 Farm Labour 劳动力投入

		Number 人数	Days 年工作天	season 季节	hrs/day 天工作小时	Pay 工资
家庭成员	Men 男					
	Women 女					
	Child 儿童					
全职员工	Men 男					
	Women 女					
**Part-time** 兼职员工						

Section 8 Feed fertilizer Inputs

Table 8.1 饲料投入

**Feed Category** 饲料种类	**FeedType** 饲料类型	**Weight (t)**总量（吨）	**Cost** 价格	**eFCR** 经济饲料转化率	**Protein %** 蛋白含量%	**Producer** 生产商/品牌	**Feed method** 投喂方式

**Commercial pellet feed** 商业颗粒饲料							
**On-farm pellet** 总的养殖场自配颗粒料							
饲料原料 **1** 注明：							
饲料原料 **2** 注明：							
饲料原料 **3** 注明：							
饲料原料 **4** 注明：							

Table 8.2 Fertiliser 肥料

**FertType** 肥料种类	**TotalUse** 使用总量	**Units** 单位	**Cost** 总成本

Section 9. Stock Losses and Disease

Table 9.1 损失 **Stock losses** 排序

**LossCauseCD** 造成损失的原因	重要性排序
Don’t know 不知道	
Feed Quality 饲料质量	
Water Quality 水质	
Disease/Parasite 病害/寄生虫	
ExtremeWeather 极端天气	
Escape 逃脱	
Cull 挑选剔除	
Poor quality seed 质量差的苗种	
Poaching 盗窃	
Predation 捕食	

Table 9.2 有哪些疾病症状

	**Species** 养殖品种	**SymptomCD** 症状			
**PrimarySpecies** 主要品种					
**SecondarySpecies** 次要品种					

Table 9.3 any measure to prevent mortality？ 采取什么措施阻止损失？

Section 10 Water Management 水的管理

Source&	**Main water source CD** 水的主要来源	
	**Secondary water source CD** 水的次要来源	
	**WaterStorageTreatMethodCD** 用水的处理方法	

	**Recirculate&ReuseWaterCD** 再循环或重复利用的水*	No 无[ ]<br>Partially 部分[ ]<br>Fully 全部[ ]

Table 10.2。Discharge 水排放

Discharge	**Max water replacement (@max density)**换水最大量(在养殖密度最大时)	%<br>[ ]<br>Depth[ ] cm		
	**Water replacement freq** 换水频率			
	**Main water discharge to CD** 主要的排水去向			
	**Effluent treatment method CD** 废水处理方法			

Table 10.3 Sediment removal 沉积物去除和水质

**removal frequency** 沉积物去除频率	
**Fate of sedimentCD** 沉积物的去向	

Section 11 Other Inputs **第 11 部分**其它的投入

Table 11.1 土壤/水处理—杀虫剂、消毒剂、微生态制剂等的使用

**SubstanceCatCD**	**TotalUse** 总的用量	**UseUnitsCD** 用量单位	**Protective measures CD** 个人防护措施	**TotalCost** 总的成本
Quicklime 生石灰				
强氯精				

Table 11.3 疾病治疗和饲料添加剂—浸泡还是加入饲料中作为药饵

**Commercial name** 商品名	**Substance CD** 主要成分	**Amount Perapplication** 每次用量	**AmountUnitsCD** 用量单位(填重量或体积单位)	Application Frequency 使用频率	**Total Cost** 总成本	**Cost Units CD** 成本单位

Section 12 Energy use Over the last complete culture cycle.

Table Table 12.1 Energy Sources 能量来源

**Energy SourceCD**	**Rank** 排序	**Total** 总量	**UnitsCD** 单位	**TotalCost** 总成本
Grid electricity 电能				
Diesel 柴油				
Gasoline 汽油				

Section 13 Financial assets, public transfers, insurance

For the respondents household 针对受访者家庭

AssetCatCD	AssetTypeCD 类型	Y/N	细节
Technical 技术	Tech assist Govt 政府部门的技术帮助		
	Tech assist Uni's/Res Inst's 大学或研究所帮助		
	Supply companies 公司		
Subsidy	Public subsides/ grants 政府补贴		
Insurance	Aquaculture insurance 水产养殖保险		

Section 14 Trends 趋势

What changes of your production system in last five years
在过去 **5** 年中，养殖系统有没有变化？

Farming area 养殖面积	
Infrastructure 基础设施	
Species/strain 品种	
Stocking density 养殖密度	
Overall production 产量	

What changes of your input in last five years
在过去 **5** 年中，投入品有没有变化？

	Type /brand 种类/品牌	Quality 数量	Price 价格	eFCR	Feeding methods
Feed 饲料					
Fertilizer 肥料				\	\
Seed 苗种				\	\
Labor 劳动力				\	\

What changes of the environment in last five years
在过去 **5** 年中，环境有没有变化？

Weather 天气	

Water quality 水质	

In this area, any above mentioned changes in other farms?
在过去 **5** 年中，本区域其他养殖户有没有显著的、上述提到的变化？

Section 15 Visual Observation 目测观察

Q15a 被采访人是否合作？是 [ ]/否 [ ]

Q15b 对农民的行为和态度作一下评价

Q15c 写下姓名的受访者问答了所有的问题了吗？是[ ] 否[ ]

Q15d 如果没有的话，什么原因，谁回答的哪个问题？

！！！GPS recorded GPS 已记录？ 是**[ ] ！！！**
